# Predicting future risk of type 1 and type 2 diabetes after gestational diabetes mellitus using polygenic scores: a cohort study

**DOI:** 10.1007/s00125-026-06824-0

**Published:** 2026-08-04

**Authors:** Isabella L. Jørgensen, Peter Damm, Mette K. Andersen, Pauline K. Reim, Lana R. F. Madsen, Mie K. W. Crusell, Louise Kelstrup, Lene Ringholm, Elisabeth R. Mathiesen, Jeannet Lauenborg, Torben Hansen, Anne C. B. Thuesen

**Affiliations:** 1 Center for Pregnant Women with Diabetes, Rigshospitalet, Copenhagen, Denmark; 2 Department of Gynaecology, Fertility and Obstetrics, Rigshospitalet, Copenhagen, Denmark; 3 Department of Clinical Medicine, Faculty of Health and Medical Sciences, University of Copenhagen, Copenhagen, Denmark; 4 Novo Nordisk Foundation Center for Basic Metabolic Research, Faculty of Health and Medical Sciences, University of Copenhagen, Copenhagen, Denmark; 5 Department of Obstetrics and Gynaecology, Zealand University Hospital, Roskilde and Nykøbing Falster, Denmark; 6 Department of Obstetrics and Gynaecology, Amager and Hvidovre Hospital, Hvidovre, Denmark; 7 Department of Obstetrics and Gynaecology, Herlev and Gentofte Hospital, Herlev, Denmark; 8 Department of Nephrology and Endocrinology, Rigshospitalet, Copenhagen, Denmark

**Keywords:** Gestational diabetes mellitus, Polygenic score, Pregnancy, Type 1 diabetes, Type 2 diabetes

## Abstract

**Aims/hypothesis:**

Women with previous gestational diabetes mellitus (GDM) have an increased risk of developing both type 1 and type 2 diabetes. However, predicting the risk on a personal level is challenging. Polygenic scores (PGSs), which capture an individual's genetic susceptibility to disease, may improve risk prediction. We aimed to examine the utility of PGSs for type 1 diabetes (T1D-PGS) and type 2 diabetes (T2D-PGS) to predict overt diabetes in women with previous GDM, with a median follow-up time of 30 years after index pregnancy.

**Methods:**

We conducted an observational study including 370 women diagnosed with GDM between 1978 and 1996. Development of type 1 and type 2 diabetes was identified using national registry data comprising ICD-10 diagnosis codes on diabetes until 2019, combined with a glucose tolerance test performed in 2000–2002. The predictive ability of T1D-PGS and T2D-PGS and clinical risk factors during index pregnancy for type 1 and type 2 diabetes, including maternal age at delivery, pre-pregnancy BMI, fasting plasma glucose, insulin treatment and family history of diabetes, was evaluated using receiver operating characteristic area under the curve (AUC).

**Results:**

During a median follow-up of 30 years, 200 (54.1%) women were diagnosed with diabetes: 30 (8.1%) with type 1 diabetes, 157 (42.4%) with type 2 diabetes and 13 (3.5%) carried monogenic diabetes variants. The mean T1D-PGS was higher in women with type 1 diabetes compared with those without (13.6 vs 11.5, *p*<0.001), while the T2D-PGS was similar between women with and without type 2 diabetes (52.9 vs 52.8, *p*=0.16). The T1D-PGS and clinical risk factors during pregnancy were discriminative of type 1 diabetes with AUCs of 0.788 and 0.839, respectively, while a combined model yielded an AUC of 0.887. The T2D-PGS and clinical risk factors during pregnancy yielded AUCs of 0.539 and 0.745, respectively. The combined model had an AUC of 0.752.

**Conclusions/interpretation:**

In women with previous GDM, the T1D-PGS showed good predictive ability for the development of type 1 diabetes, especially when combined with clinical risk factors during pregnancy, and may have potential to guide targeted follow-up and early treatment interventions. The T2D-PGS showed no predictive ability for type 2 diabetes.

**Graphical Abstract:**

**Figure:**
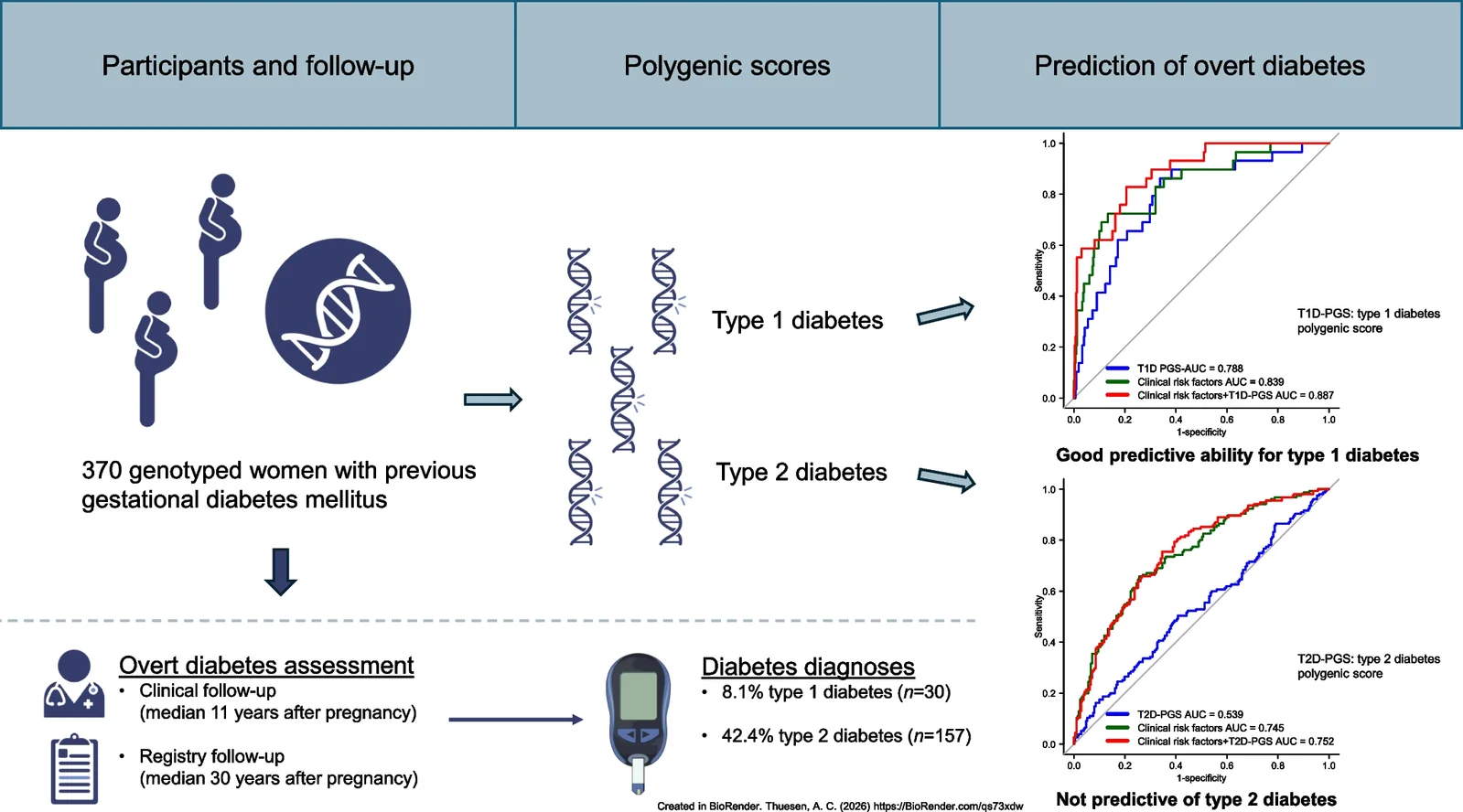

**Supplementary Information:**

The online version contains peer-reviewed but unedited supplementary material available at https://doi.org/10.1007/s00125-026-06824-0.

**Figure:**
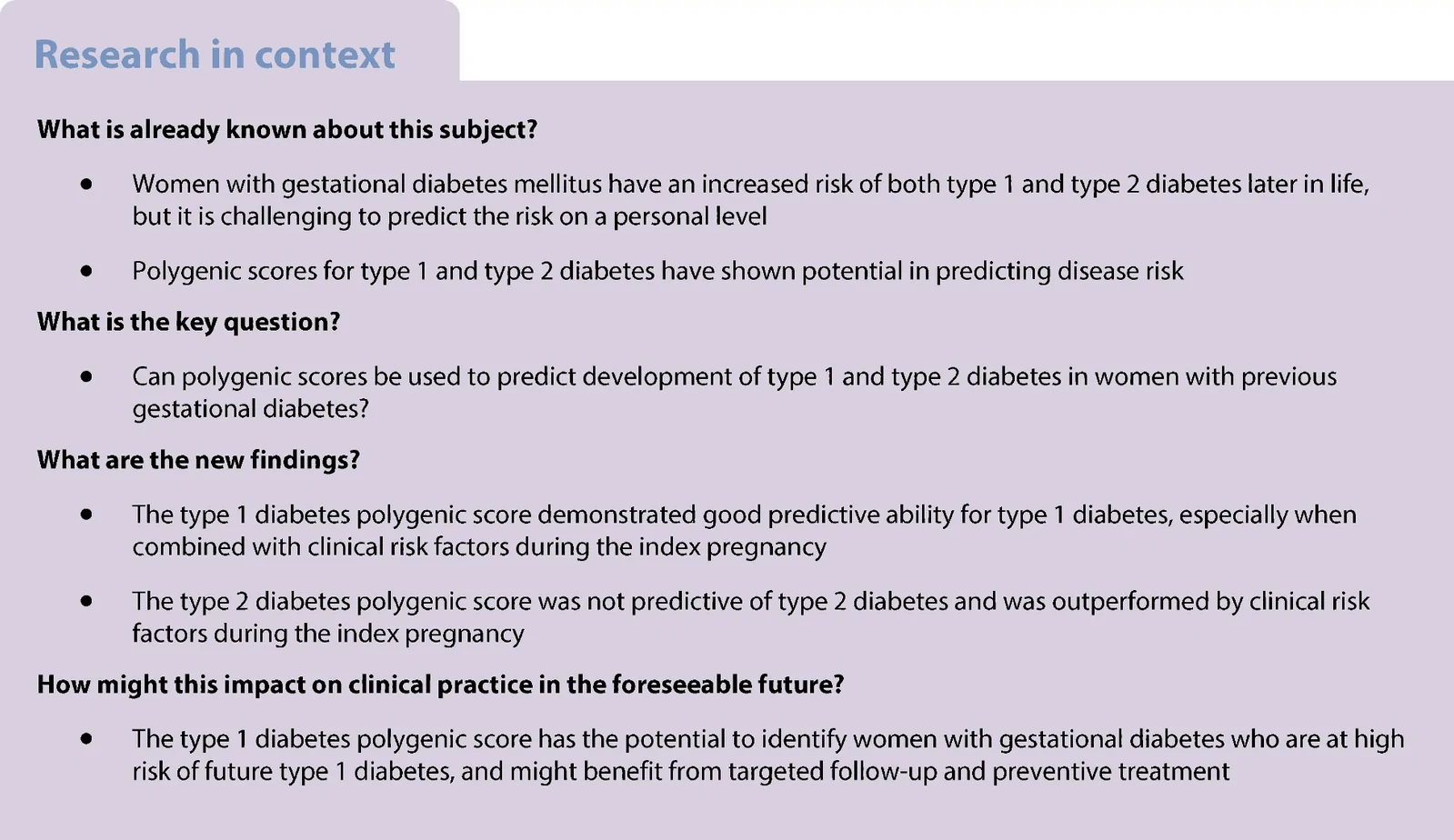

## Introduction

Gestational diabetes mellitus (GDM) is characterised by abnormal glucose tolerance diagnosed or recognised for the first time during pregnancy [1]. The prevalence of GDM has increased over the past decades, making it one of the most common pregnancy complications, affecting around 6–15% of pregnancies worldwide [2]. Women diagnosed with GDM have a markedly increased life-long risk of both type 1 and type 2 diabetes [3]. The prevalence of type 1 diabetes after GDM has been reported to be 4–7% in European follow-up studies [4–6], and early identification is critical due to potentially life-threatening acute complications, such as ketoacidosis [7, 8]. Development of type 2 diabetes occurs in up to 50% of women with previous GDM [5, 6].

While population-level clinical risk factors for subsequent diabetes, such as pre-pregnancy obesity, early GDM diagnosis, insulin treatment for GDM and severity of hyperglycaemia [5, 6, 9], as well as the presence of autoantibodies for type 1 diabetes [10, 11], are well-established, accurate risk prediction at the individual level remains challenging. However, with the rising prevalence of GDM, there is an increasing need for tools that enable personalised risk assessment for the development of type 1 or type 2 diabetes after GDM for more efficient use of health resources.

The aetiologies of type 1 and type 2 diabetes are multifactorial, including both genetic and environmental factors. The genetic contribution to diabetes risk involves multiple genes, and the cumulative contribution of genetic variation can be expressed as a single-value polygenic score (PGS), which has potential as a risk prediction tool [12–14].

The genetic contribution to type 1 diabetes is strong and predominantly associated with genes within the human leukocyte antigen (HLA) region [15]. PGS for type 1 diabetes (T1D-PGS) has proven to be highly discriminative of type 1 diabetes, especially when combined with clinical risk factors [13, 14, 16–20], but the predictive ability of T1D-PGS in women with previous GDM remains unexplored. However, with new promising treatment options available to delay disease progression of type 1 diabetes [8, 21], identifying high-risk individuals within the GDM population may hold clinical importance.

The genetic contribution to type 2 diabetes risk includes more than 1000 genetic variants across the genome, most with small effects on disease risk [12, 22]. PGS for type 2 diabetes (T2D-PGS) has demonstrated improvements in predicting type 2 diabetes in women with previous GDM compared with clinical risk factors alone [23, 24], indicating a potential for enhancing risk stratification. However, previous studies included women diagnosed using different GDM criteria, assessed clinical risk factors at varying time points or had relatively short follow-up periods, limiting the ability to predict late-onset type 2 diabetes. This underscores the need to assess the predictive ability of a T2D-PGS in a cohort with standardised diagnostic criteria for GDM, available risk factors during pregnancy and a long follow-up period.

We hypothesised that T1D-PGS and T2D-PGS can be used to predict future diabetes development in women with GDM. We aimed to evaluate the predictive ability of the PGSs for overt diabetes in women with previous GDM, with a median follow-up of 30 years after index pregnancy.

## Methods

### Study design and population

**Fig. 1:**
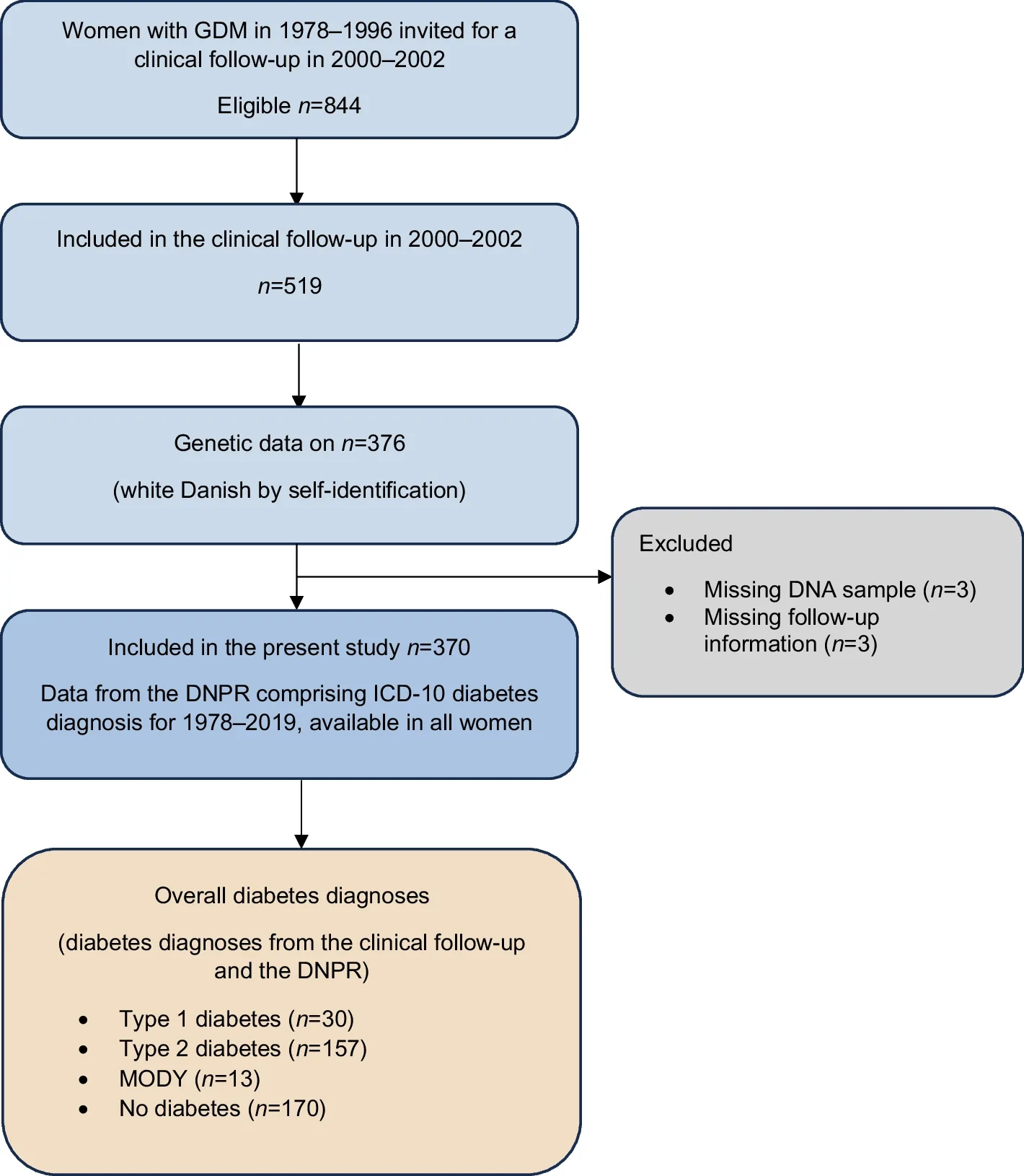
Flowchart of inclusion of 370 women with previous GDM and diabetes diagnoses. Women with type 1 diabetes (*n*=30) were all classified based on a type 1 diabetes diagnosis (ICD-10: DE10) in the DNPR, and all (*n*=30 [100%]) had developed diabetes at the clinical follow-up; women with type 2 diabetes (*n*=157) were classified based on a diagnosis of type 2 diabetes (ICD-10: DE11), unspecified diabetes (ICD-10: DE14) or other diabetes (ICD-10: DE13) in the DNPR (*n*=136 [86.6%]), as were women without a registered diabetes diagnosis in the DNPR who instead met the criteria for type 2 diabetes at the clinical follow-up (*n*=21 [13.4%]); MODY (*n*=13) was diagnosed based on the presence of disease-causing variants in MODY genes [34], and these included seven glucokinase (*GCK*)-MODY, five hepatocyte nuclear factor-4-α (*HNF4A*)-MODY and one hepatocyte nuclear factor-1-α (*HNF1A*)-MODY; no diabetes was classified as women who did not have a diabetes diagnosis in the DNPR and did not meet the diabetes criteria at the clinical follow-up

The study population included 370 women diagnosed with GDM from 1978 to 1985, or 1987 to 1996, who had participated in a clinical follow-up study between 2000 and 2002 at the Center for Pregnant Women with Diabetes, Rigshospitalet, Copenhagen, Denmark (Fig. 1) [6, 25, 26]. The GDM diagnosis was based on a selective screening strategy in case of the presence of risk factors, including family history of diabetes, pre-pregnancy BMI >27 kg/m^2^, previous macrosomia (birthweight ≥4500 g), glucosuria, previous GDM, age ≥35 years or increased fasting blood glucose [6].

Between 1978 and 1985, women were diagnosed with GDM based on a 3 h 50 g OGTT, and between 1987 and 1996 with a 3 h 75 g OGTT. Test criteria changed in 1986, which is why this year is not included in the study. The OGTTs were diagnostic of GDM, consistent with the diagnostic criteria at the time, if two or more of seven values exceeded 3 standard deviations (SD) above the mean for a group of non-pregnant women with normal weight and no family history of diabetes (specific diagnostic threshold values for the 50 g and 75 g OGTTs are given in electronic supplementary material [ESM] Table 1) [6]. Information on index pregnancy was obtained from medical records and included pre-pregnancy BMI, maternal age at delivery, risk factors for GDM screening, glucose values from the diagnostic OGTT, GDM treatment and gestational age at diagnostic OGTT and at delivery.

A detailed description and comparison of index pregnancies between participants and non-participants in the clinical follow-up has been reported previously [6]. In brief, participants were of similar age but had lower pregestational BMI and diabetes prevalence postpartum compared with non-participants. The present cohort, restricted to participants with available DNA [26], included only self-reported white Danes and had fewer insulin-treated women than in the background GDM population. We did not have information on socioeconomic factors.

The study was approved by the Danish Committee on Health Research Ethics (J.nr: 01-048/99 and 11-082/01) and the Legal Department of the Capital Region (P-2022-560)*.* Written informed consent was obtained from all participants.

### Clinical follow-up

Between 2000 and 2002, the 370 women participated in a clinical follow-up study that aimed to examine the incidence and predictors of abnormal glucose tolerance in women with previous GDM. The study has previously been described in detail [6, 26]. Briefly, women without known diabetes were examined with a 2 h 75 g OGTT following a 10 h fast. Venous plasma glucose and C-peptide levels at fasting and at 30 and 120 min were measured, as well as serum glutamic acid decarboxylase autoantibodies (GADA). Diabetes criteria were fasting plasma glucose (FPG) ≥7.0 mmol/l or 2 h plasma glucose ≥11.1 mmol/l. Women reporting known insulin-treated diabetes at follow-up were examined with a glucagon test. Fasting C-peptide was measured, followed by intravenous injection of 1 mg glucagon (Glucagen, Novo Nordisk), whereafter C-peptide levels were reassessed after 6 min. Type 1 diabetes was classified in women with fasting serum C-peptide <300 pmol/l and 6 min C-peptide <600 pmol/l. Plasma glucose was analysed on a Cobas Mira analyser (Roche, Mannheim, Germany). Serum C-peptide was analysed on an AutoDelfia (Wallac/PerkinElmer, Turku, Finland). GADA were detected by radioimmunoassay (Diamyd Anti-GAD-65 RIA; Diamyd Diagnostics, Stockholm, Sweden).

Weight and height were measured, and BMI was calculated as weight (kg)/height (m)^2^. Information on family history of diabetes was obtained from questionnaires.

### Registry data

In Denmark, data on healthcare services are collected prospectively and stored in national registries linked to an individual’s unique identification number [27]. We used data from the Danish National Patient Registry (DNPR) comprising the 10th revision of the International Classification of Diseases (ICD-10) diagnosis codes from hospital contacts (as both primary and secondary diagnoses), to assess diabetes diagnoses [28], and from the Danish Register of Causes of Death to determine follow-up time after index pregnancy [29]. Data were provided by Statistics Denmark and the Danish Health Data Authority.

### Genetic data and generation of PGSs

Participants were genotyped using the Infinium Global Screening Array-24 v3.0 BeadChip (Illumina, San Diego, United States) and imputed using the HRC and 1KG reference panels [30, 31].

We calculated a T1D-PGS based on 67 genetic variants across known type 1 diabetes loci, including HLA interaction terms. This PGS was previously generated and validated in participants from the Type 1 Diabetes Genetic Consortium and UK Biobank [13].

We also computed a T2D-PGS using PRSice version 2.3.5 [32]. The T2D-PGS was derived from summary statistics of a large-scale genome-wide association study in participants of primarily European ancestry [33] and validated in the UK Biobank [12]. The T2D-PGS included 6,917,436 genetic variants (https://www.pgscatalog.org/score/PGS000014), of which 6,519,214 were available for our analysis.

### Outcomes

The primary outcomes were development of type 1 or type 2 diabetes, assessed based on diabetes diagnoses from the clinical follow-up between 2000 and 2002, and ICD-10 diagnosis codes from the DNPR, from index pregnancy until 31 December 2019, or death, whichever occurred first. The diagnosis codes included ICD-10: DE10 (type 1 diabetes), DE11 (type 2 diabetes), DE13 (other diabetes) and DE14 (unspecified diabetes). Individuals with type 1 diabetes were followed in secondary care, where reporting to the registries is mandatory. All women registered with type 1 diabetes (DE10) in the DNPR were classified as such in our study, with disease onset defined as the date of the first registration. Women registered with type 2 diabetes (DE11), unspecified (DE14) or other diabetes (DE13) were classified as having type 2 diabetes. Most individuals with type 2 diabetes in Denmark are managed in primary care, from which diagnoses are not reported to national registries. For the same reason, it was not possible to determine the time of type 2 diabetes onset. To minimise underreporting of type 2 diabetes, women that did not have a diabetes diagnosis in the DNPR but met the criteria for type 2 diabetes at the clinical follow-up between 2000 and 2002 were also classified as having type 2 diabetes. In addition, the presence of rare genetic variants causing maturity-onset diabetes of the young (MODY) among the women in our cohort has previously been reported [34]. Women with a diabetes diagnosis who carried pathogenic or likely pathogenic variants, or variants of uncertain significance leaning pathogenic, according to ClinGen Monogenic Diabetes Expert Panel specifications [31], were classified as having MODY to prevent misclassification. Women who did not meet the diabetes criteria at the clinical follow-up and did not have a diabetes diagnosis in the registries were classified as not having diabetes.

### Statistical analysis

Data are given as mean ± SD or median (interquartile range [IQR]) for numerical variables depending on normal distribution, and as number (%) for categorical variables. Student's *t* test was used to compare PGS values. Logistic regression was used to determine odds ratio (OR) for the association between high type 1 diabetes genetic risk and type 1 diabetes. High type 1 diabetes risk was defined based on a previously established cutoff value of >14.6, corresponding to the 50th percentile T1D-PGS for European individuals with type 1 diabetes in the UK Biobank [13]. We considered two-sided *p* values <0.05 to be statistically significant. To determine the predictive ability of the PGSs for development of type 1 and type 2 diabetes, we used receiver operating characteristic (ROC) area under the curve (AUC) based on logistic regression models, with complete follow-up time (from delivery to 31 December 2019 or death) included as a covariate using linear, logarithmic and quadratic terms to account for differences in observation time. For type 1 diabetes, we also performed a sensitivity analysis of the prediction within the first 10 years after index pregnancy, since most type 1 diabetes after GDM occurs within this period [5]. We also incorporated clinical risk factors for diabetes obtained during index pregnancy, including maternal age at delivery, pre-pregnancy BMI, FPG at diagnostic OGTT, insulin treatment and family history of diabetes, along with GADA measurements from the clinical follow-up a median of 11 years after index pregnancy. Differences in AUC between the T1D-PGS and clinical risk factor models were compared using DeLong’s test. Youden’s index (sensitivity + specificity − 1) was used to determine Youden’s maximum, which optimises the sum of sensitivity and specificity. To assess the association between the PGSs and overt diabetes development, the cohort was divided into two groups: type 1 diabetes vs no type 1 diabetes (type 2 diabetes, MODY and no diabetes), and type 2 diabetes vs no type 2 diabetes (type 1 diabetes, MODY and no diabetes). Missing values are reported in table and figure legends. Statistical analyses were conducted in R version 4.5.1 (R Project for Statistical Computing; https://www.r-project.org).

## Results

### Pregnancy characteristics and diabetes development

Pregnancy characteristics of the 370 women with previous GDM are presented in Table 1. The clinical follow-up between 2000 and 2002 was conducted at a median of 11 years (range: 4–23 years) after index pregnancy, while the median registry follow-up time after index pregnancy was 30 years (range: 23–42 years). In total, 200 women (54.1%) developed diabetes: 30 women (8.1%) were diagnosed with type 1 diabetes, 157 women (42.4%) with type 2 diabetes, 13 women (3.5%) carried putatively causal MODY variants, while 170 women (45.9%) did not have a diabetes diagnosis. A flowchart illustrating the inclusion and diabetes classification is presented in Fig. 1. Baseline characteristics according to diabetes development are given in ESM Table 2.

**Table 1:** Pregnancy characteristics of 370 women with GDM diagnosed between 1978 and 1996

Characteristic	Value
Maternal age at delivery (years)	31.4±5.3
Pregestational BMI (kg/m^2^)	24.4 (21.4–29.7)
Gestational age at GDM diagnosis (weeks + days)	32+4 (28+1–35+4)
Gestational age at delivery (weeks + days)	39+0 (38+0–39+5)
FPG at diagnosis (mmol/l)	5.2 (4.6–5.7)
Treatment^a^	
Diet	345 (93.8)
Insulin	23 (6.2)

Data are given as median (IQR), mean ± SD or *n* (%). Data were available on >95% of participants. All were white Danish by self-identification

^a^Two women had missing treatment data

### T1D-PGS and prediction of type 1 diabetes during the median 30 year follow-up after index pregnancy

**Fig. 2:**
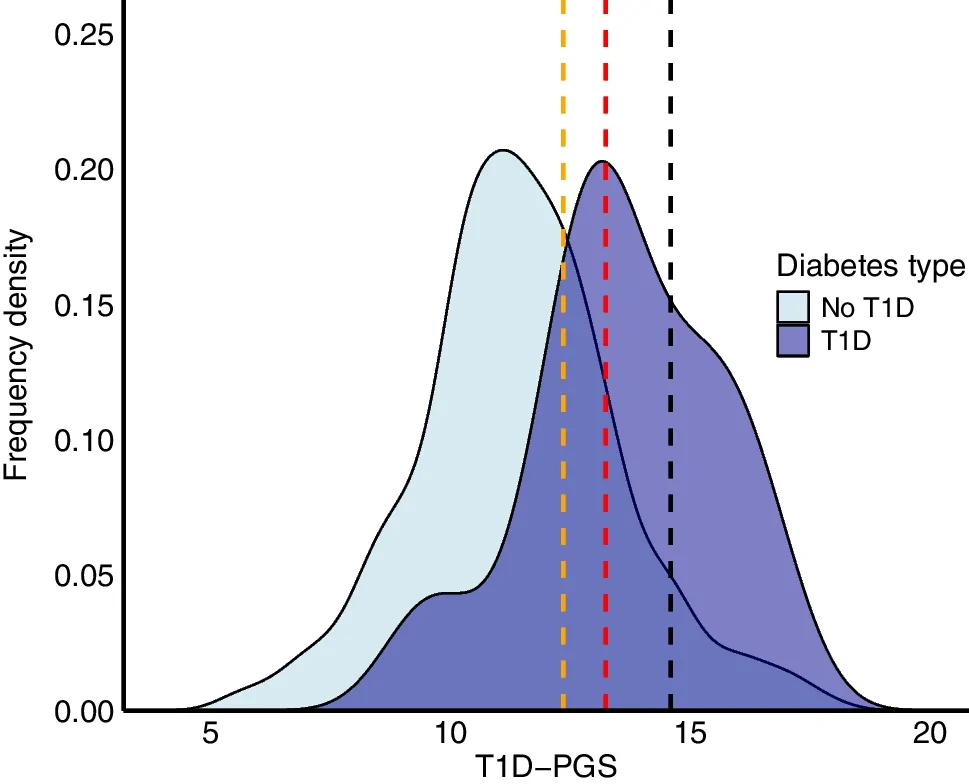
T1D-PGS distribution in women with previous GDM with and without type 1 diabetes. Women categorised as no T1D had either no diabetes diagnosis, type 2 diabetes or MODY. The PGS distribution was similar among these three groups (Fig. 3a). The orange line represents the maximum Youden’s index, the red line represents the 50th percentile PGS among women with type 1 diabetes in our study, while the black line represents the 50th percentile PGS in European type 1 diabetes cases in the UK Biobank [13]. The T1D-PGS was available in 365 women. T1D, type 1 diabetes

**Fig. 3:**
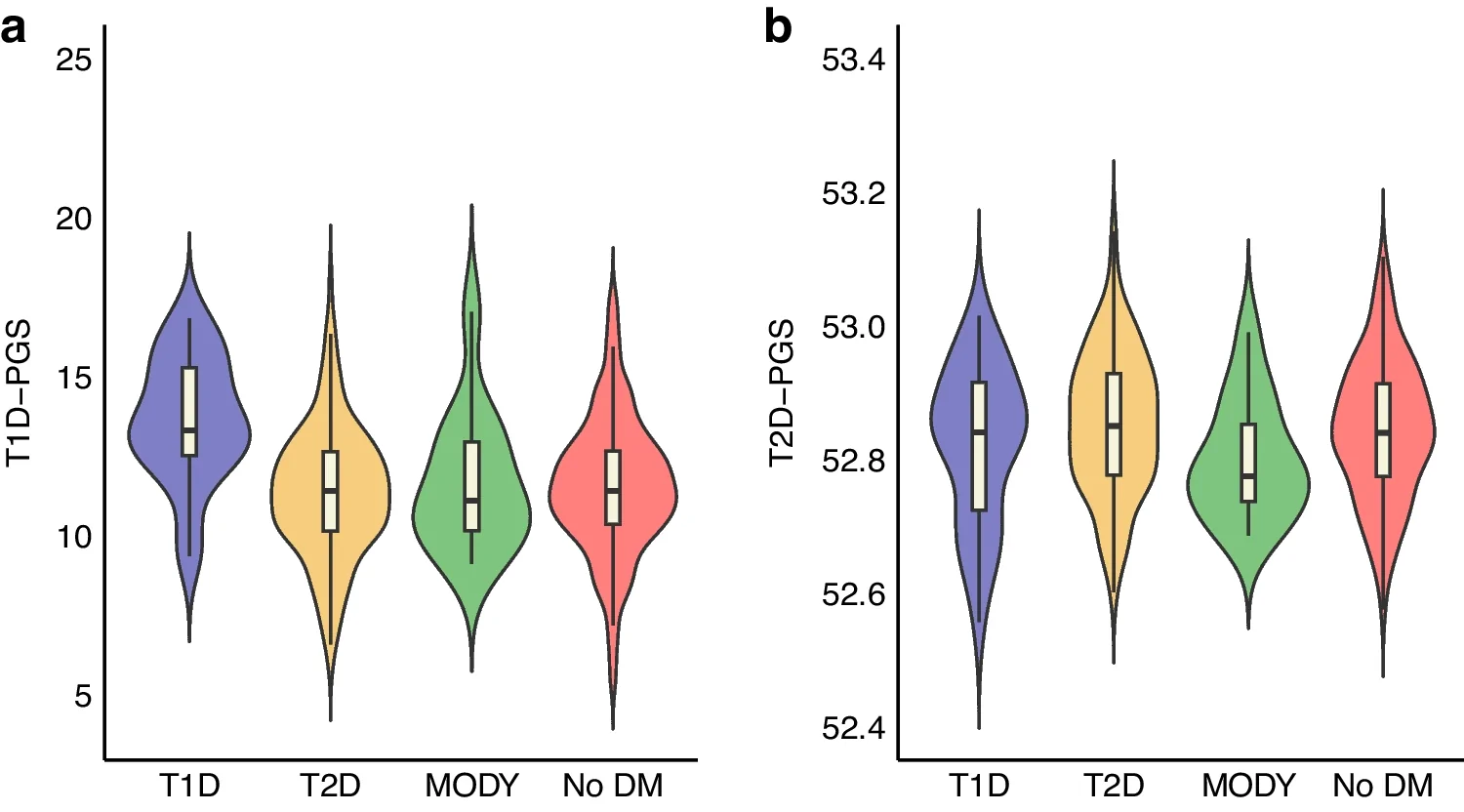
(**a**, **b**) Violin plots of the T1D-PGS and T2D-PGS according to diabetes development in women with previous GDM. T1D-PGS (**a**) and T2D-PGS (**b**) distributions in women with previous gestational diabetes according to diabetes development, with a median follow-up of 30 years after index pregnancy. The central box represents the IQR, while the line inside indicates the median. MODY types included seven glucokinase (*GCK*)-MODY, five hepatocyte nuclear factor-4-α (*HNF4A*)-MODY and one hepatocyte nuclear factor-1-α (*HNF1A*)-MODY. The T1D-PGS was available in 365 women, and the T2D-PGS was available in all 370 women. No DM, no registered diabetes diagnosis; T1D, type 1 diabetes; T2D, type 2 diabetes

**Fig. 4:**
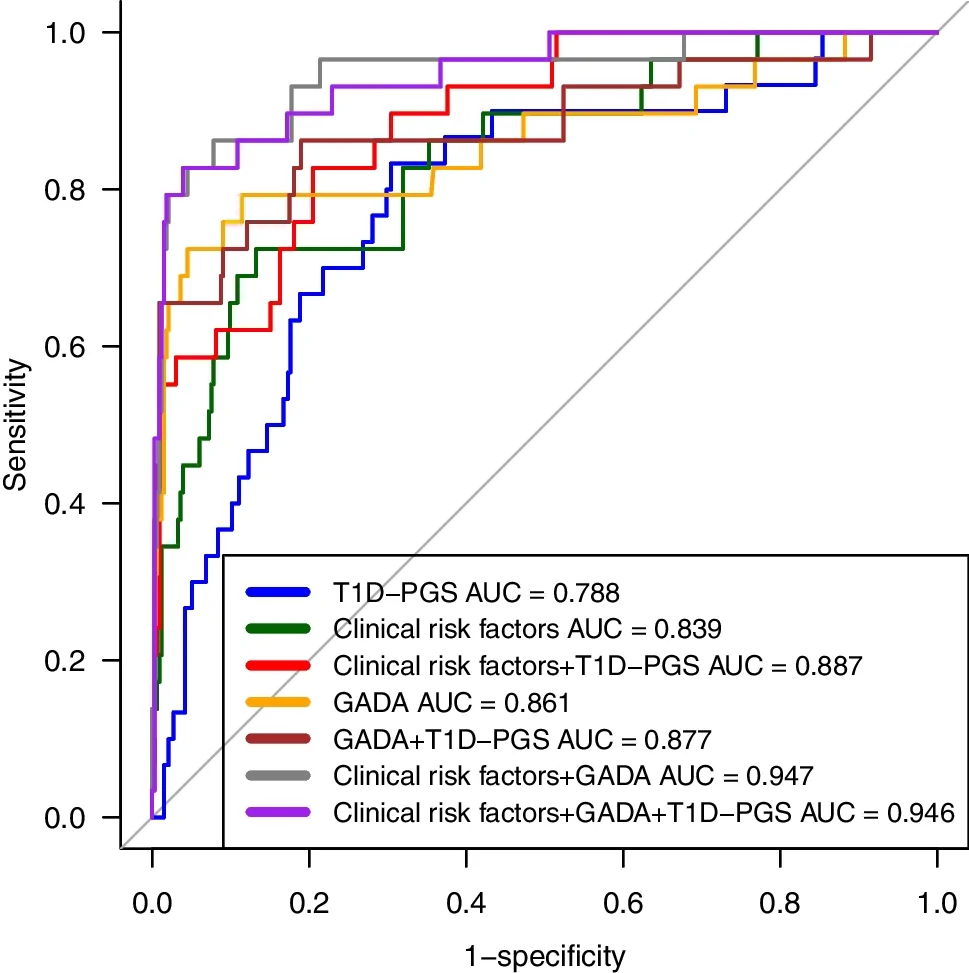
ROC AUC for prediction of type 1 diabetes in women with previous GDM with a median follow-up of 30 years after index pregnancy using a PGS and clinical risk factors from pregnancy. Clinical risk factors during index pregnancy included maternal age at delivery, pre-pregnancy BMI, FPG at GDM diagnosis, insulin treatment and family history of diabetes. GADA were measured at the clinical follow-up at a median of 11 years after index pregnancy. The models are adjusted for follow-up time. The analysis included 361 women with complete data (five had missing T1D-PGS and four had missing clinical data). Of these, 29 had type 1 diabetes, and 332 did not have type 1 diabetes

Type 1 diabetes was registered for the first time in the DNPR a median of 5 years (range: 0–22 years) after index pregnancy. The mean T1D-PGS in the 30 women that developed type 1 diabetes was significantly higher than in the remaining women (13.6 vs 11.5, *p*<0.001) (Fig. 2). The T1D-PGS distribution was similar in women diagnosed with type 2 diabetes and MODY and with no diabetes diagnosis (Fig. 3a). To explore the potential clinical use of the T1D-PGS, we tested a cutoff value corresponding to the 50th percentile T1D-PGS (>14.60) in a British type 1 diabetes population [13]. This cutoff gave an OR of 7.16 (95% CI 2.74, 18.68, *p*<0.001) for type 1 diabetes, when adjusted for maternal age at delivery, pre-pregnancy BMI and follow-up time, with a sensitivity of 33% and specificity of 93%. The 50th percentile T1D-PGS in women with type 1 diabetes (50% sensitivity) in our cohort was 13.32, with a specificity of 83%. Youden’s maximum was observed at a T1D-PGS value of 12.36, resulting in a sensitivity of 83% and specificity of 70%. The T1D-PGS alone was discriminative of type 1 diabetes with an AUC of 0.788 (95% CI 0.702, 0.875), while clinical risk factors during index pregnancy were discriminative with an AUC of 0.839 (0.758, 0.921) (AUC difference = 0.051 [−0.168, 0.067], *p*=0.40). When the T1D-PGS and clinical risk factors were combined, the AUC was 0.887 (0.828, 0.946) (AUC difference from clinical risk factors only = 0.048 [−0.003, 0.098], *p*=0.06) (Fig. 4). GADA measured at the clinical follow-up at a median of 11 years after index pregnancy were discriminative of type 1 diabetes with an AUC of 0.861 (0.767, 0.955), while GADA combined with the T1D-PGS yielded an AUC of 0.877 (0.791, 0.963). The strongest predictive ability was observed when combining clinical risk factors from pregnancy with GADA measured at the clinical follow-up, at a median of 11 years after pregnancy, yielding an AUC of 0.947 (0.897, 0.996). The inclusion of the T1D-PGS did not further improve prediction with an AUC of 0.946 (0.901, 0.990) (Fig. 4).

### T1D-PGS and prediction of type 1 diabetes within 10 years after index pregnancy

**Fig. 5:**
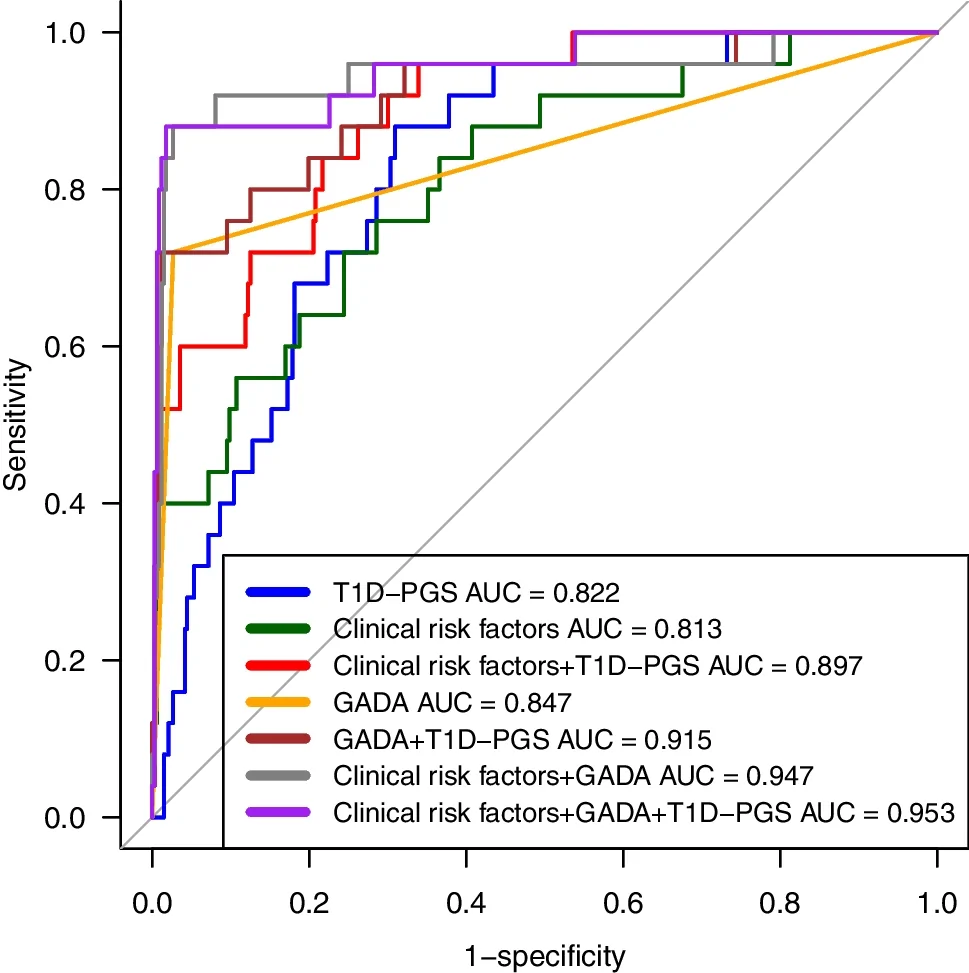
ROC AUC for prediction of type 1 diabetes development within 10 years after index pregnancy using a PGS and clinical risk factors from pregnancy. Of the 30 women with previous GDM who developed type 1 diabetes, 26 (86.7%) had a registered diabetes diagnosis within 10 years after index pregnancy. Clinical risk factors during index pregnancy included maternal age at delivery, pre-pregnancy BMI, FPG at GDM diagnosis, insulin treatment and family history of diabetes. GADA were measured at the clinical follow-up at a median of 11 years after index pregnancy. The analysis included 361 women with complete data (five had missing T1D-PGS and four had missing clinical data). Of these, 29 had type 1 diabetes, and 332 did not have type 1 diabetes

Of the 30 women who developed type 1 diabetes, 26 (86.7%) had a type 1 diabetes diagnosis in DNPR within 10 years after index pregnancy. During this period, the T1D-PGS alone predicted type 1 diabetes with an AUC of 0.822 (0.751, 0.893), and clinical risk factors during index pregnancy with an AUC of 0.813 (0.723, 0.904) (AUC difference = 0.009 [−0.109, 0.126], *p*=0.89), while combined the AUC was 0.897 (0.840, 0.955) (AUC difference from clinical risk factors only = 0.084 [0.022, 0.146], *p*=0.008). The strongest discriminative ability was observed when combining the T1D-PGS, clinical risk factors during index pregnancy and GADA from clinical follow-up, yielding an AUC of 0.953 (0.904, 1) (Fig. 5).

### T2D-PGS and prediction of type 2 diabetes during the median 30 year follow-up after index pregnancy

**Fig. 6:**
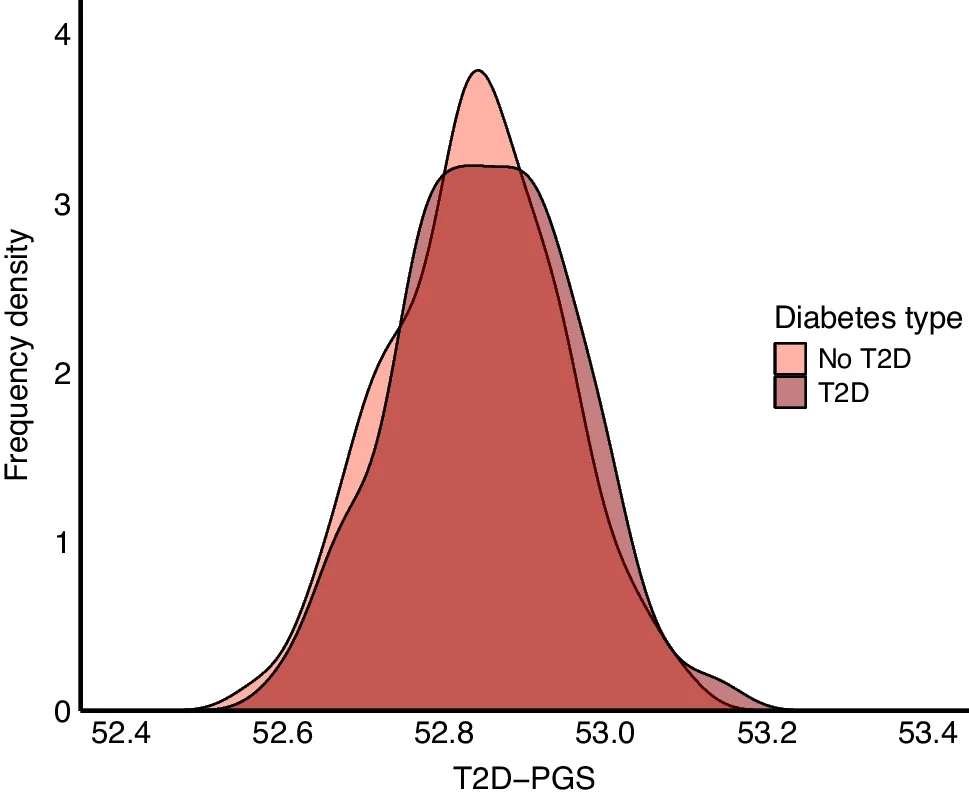
T2D-PGS distribution in women with previous GDM with and without development of type 2 diabetes. Women categorised as no T2D had either no diabetes diagnosis, type 1 diabetes or MODY. The PGS distribution was similar among all diabetes groups (Fig. 3b). T2D, type 2 diabetes

**Fig. 7:**
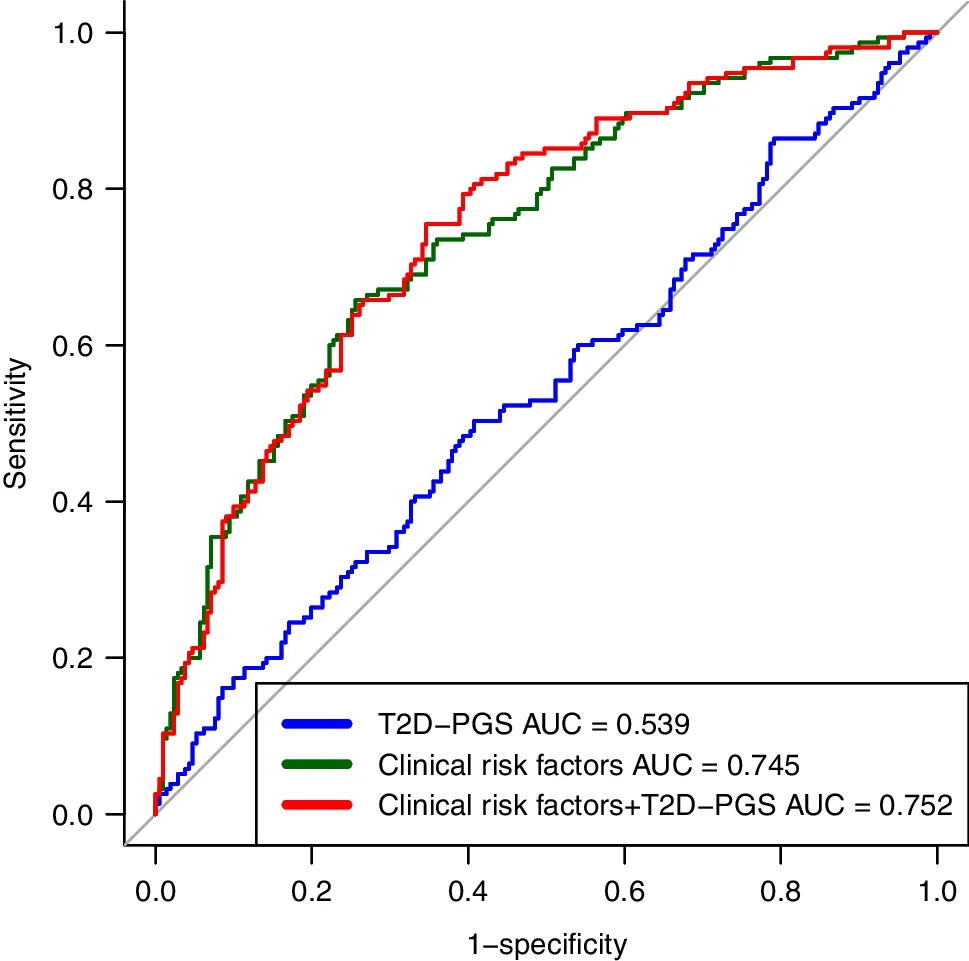
ROC AUC for prediction of type 2 diabetes with a median follow-up of 30 years after index pregnancy using a PGS and clinical risk factors from pregnancy. Clinical risk factors during index pregnancy included maternal age at delivery, pre-pregnancy BMI, FPG at GDM diagnosis, insulin treatment and family history of diabetes. The models are adjusted for follow-up time. The analysis included 366 women with complete data (four had missing clinical data). Of these, 155 had type 2 diabetes, and 211 did not have type 2 diabetes

The mean T2D-PGS among women with type 2 diabetes (52.8) did not differ from the mean T2D-PGS (52.9) among those without type 2 diabetes (*p*=0.16) (Figs 3b, 6). The T2D-PGS alone was not discriminative of type 2 diabetes with an AUC of 0.539 (0.480, 0.599), while the clinical risk factors during index pregnancy were predictive of type 2 diabetes with an AUC of 0.745 (0.694, 0.796). When the T2D-PGS and clinical risk factors were combined, the AUC was 0.752 (0.703, 0.803) (Fig. 7).

## Discussion

We studied the utility of T1D-PGS and T2D-PGS in 370 women with previous GDM. We demonstrated a good ability for the T1D-PGS to predict development of type 1 diabetes, especially when combined with clinical risk factors during index pregnancy. These findings indicate that the T1D-PGS has potential to identify women with GDM at high risk of type 1 diabetes who may benefit from targeted surveillance postpartum.

The T2D-PGS was not predictive of development of type 2 diabetes, and when combined with clinical risk factors during pregnancy, it provided no substantial improvement compared with clinical risk factors alone. Overall, the variability between groups was low, which may reflect the cohort composition. Given the selective risk-factor-based screening strategy for GDM in Denmark, the GDM population probably has a higher a priori risk of diabetes than GDM populations identified through universal screening, as reflected in the relatively high prevalence of type 2 diabetes. Furthermore, temporal changes in type 2 diabetes trajectories following GDM in our cohort may also contribute to the limited predictive ability of T2D-PGS. Women diagnosed with GDM between 1987 and 1996 were older at the time of pregnancy, had a higher pregestational BMI, had more familial disposition to diabetes and progressed more rapidly to diabetes and prediabetes compared with those diagnosed earlier between 1978 and 1985 [6]. These findings suggest that the advanced maternal age and increasing prevalence of obesity over time contribute more to the development of type 2 diabetes and may thereby attenuate the relative influence of genetic factors. However, other studies have demonstrated that combining T2D-PGS with clinical risk factors in women with previous GDM improves predictive ability, with potential implications for preventive strategies [23, 35]. These discrepancies may reflect differences in clinical characteristics, including age, ethnicity, baseline metabolic risk factors and the long follow-up time among participants in our study.

To our knowledge, this is the first study to assess the utility of a T1D-PGS in women with previous GDM. Consistent with our findings, prospective studies in high-risk individuals, including children with HLA class 1 diabetes susceptibility genotypes, and individuals with a family history of type 1 diabetes who are positive for autoantibodies, have shown that higher T1D-PGS was associated with both disease progression and onset [17–20].

In this study, genetic risk was assessed using a T1D-PGS that has previously been validated in a European type 1 diabetes population within the UK Biobank, where it demonstrated strong discriminative ability of type 1 diabetes with an AUC of 0.92 and a median T1D-PGS of 14.6 among individuals with type 1 diabetes, corresponding to a sensitivity of 54% and a specificity of 97% [13]. In our study, the T1D-PGS had lower predictive performance with an AUC of 0.788. The median T1D-PGS for women who developed type 1 diabetes was 13.32, corresponding to a sensitivity of 50% and specificity of 84%. The lower median T1D-PGS and predictive capability observed in our study may reflect differences in clinical characteristics among participants. Higher genetic risk has been associated with earlier onset of type 1 diabetes [36, 37], and in our study, all participants were diagnosed in adulthood based on diagnosis codes, whereas the type 1 diabetes cases in the UK Biobank were defined using strict clinical criteria for type 1 diabetes, including development at a young age [13]. Consistently, the T1D-PGS values observed in our study align with findings from other studies of individuals with adult-onset type 1 diabetes [19, 37, 38].

In our study, the T1D-PGS, clinical risk factors during pregnancy and GADA measured at the clinical follow-up each demonstrated similar predictive ability for type 1 diabetes on their own. The highest predictive performance was achieved by combining clinical risk factors during pregnancy and GADA. It is important to note, however, that GADA were measured at the clinical follow-up, when all type 1 diabetes cases had developed, and do not necessarily reflect a prediction that would be available during pregnancy. Nevertheless, this finding underscores that if autoantibodies had been available during pregnancy, the predictive performance would probably have improved.

A major advantage of a PGS compared with clinical risk factors and biomarkers is that it remains constant throughout life and can be measured at any time point, whereas clinical predictors and biomarkers may be unavailable or vary over time. Assessing genetic risk for type 1 diabetes in women with GDM offers several clinical advantages, including targeted postpartum follow-up and more accurate disease classification. Postpartum follow-up in individuals with high T1D-PGS may include autoantibody screening, as the presence of autoantibodies has been shown to be a strong predictor of type 1 diabetes development in women with GDM [10, 11]. In other high-risk type 1 diabetes populations, autoantibody monitoring programmes have proven effective for early detection of disease progression, reducing the risk of diabetic ketoacidosis at diagnosis and allowing timely therapeutic interventions to delay the onset of overt type 1 diabetes [7, 8, 21, 39]. Another potential advantage of assessing genetic risk for type 1 diabetes is more accurate diabetes classification, as the T1D-PGS has proven effective in discriminating between type 1 and type 2 diabetes, as well as between type 1 diabetes and MODY [14, 16]. This may be particularly relevant for women with GDM, as identifying those with high risk of type 1 diabetes can be challenging due to the predominance of later-onset type 2 diabetes, combined with type 1 diabetes often being perceived as a disease occurring in childhood or adolescence [36, 40]. However, approximately half of all cases with type 1 diabetes present in adulthood, often with clinical features similar to young-onset disease, including diabetic ketoacidosis [38, 40, 41]. In this respect, the T1D-PGS may guide the diagnosis in a group with high risk of diagnostic misclassification, especially during the first decade after pregnancy, when most type 1 diabetes cases occur [5]. Consistent with this, we found that the T1D-PGS had the highest predictive performance within the first 10 years after index pregnancy.

Despite the potential advantages of using genetic screening for type 1 diabetes in women with GDM, several challenges to clinical implementation remain. A key challenge is defining clinically useful thresholds for high genetic risk. Given that type 1 diabetes is a chronic and progressive autoimmune disease with an unpredictable progression to overt disease, knowledge of high genetic risk may induce psychological stress and potentially adversely affect quality of life [42]. These considerations support the use of thresholds with high specificity to minimise false positive results. The median T1D-PGS of 13.32 among women with type 1 diabetes in our study had a moderate specificity of 84%, whereas the previously established median T1D-PGS value of 14.6 from individuals with type 1 diabetes in the UK Biobank provided a higher specificity of 93%; however, this cutoff identified only one-third of type 1 diabetes cases, highlighting the challenge of defining clinically relevant thresholds. Other challenges for clinical implementation include lack of guidelines for follow-up and monitoring of high-risk individuals. Additionally, most healthcare professionals have limited experience with interpreting and communicating PGS results, as these have primarily been used for research purposes. As such, clinical implementation would necessitate specific education and training. However, given the substantial clinical potential within this field, growing attention has been directed toward these challenges [43]. Moreover, economic considerations must be addressed, as universal genetic testing of all women with GDM would be costly. However, a more targeted approach may be warranted, focusing on those with the highest clinical risk, based on e.g. BMI and high glucose levels.

A key strength of our study is the assessment of data at multiple timepoints, including clinical risk factors during pregnancy, detailed follow-up assessments of glucose tolerance at a median of 11 years after pregnancy and registry data with ICD-10 diagnosis codes for diabetes covering up to 42 years after pregnancy. Data on clinical risk factors present at the time of pregnancy enable the assessment of the predictive ability of the T1D-PGS already at the time of pregnancy. Furthermore, the combination of the clinical examination of glucose tolerance, including measurements of C-peptide and GADA at follow-up, knowledge on MODY-predisposing genetic variants and registry data enabled thorough diagnostic assessment of diabetes development.

Our study also has limitations. A major limitation is the small number of women with type 1 diabetes in our cohort, as the condition is relatively rare. The limited sample size increases sensitivity to random variation, and validation in larger, independent cohorts is warranted. Furthermore, women were recruited based on deliveries from a single centre, and all were self-reported white Danish, which reduces the generalisability of our findings to a more unselected GDM population [44]. In addition, the T1D-PGS was developed and validated using genetic data primarily from white Europeans, and studies evaluating the utility of T1D-PGS in other ethnic groups have demonstrated varying risk estimates, raising concerns about the external validity in real-world populations of individuals with mixed ethnic backgrounds [45]. Further, as type 1 diabetes developed after pregnancy, we have no information on disease progression and cannot exclude that some women in our study may have latent autoimmune diabetes in adults (LADA), which is a slowly progressing form of autoimmune diabetes characterised by the presence of autoantibodies and a longer insulin-free period following diagnosis [46]. In Denmark, there are no specific diagnosis codes for LADA, which can result in misclassification as both type 1 and type 2 diabetes. Given that LADA typically exhibits a genetic profile intermediate between type 1 and type 2 diabetes [46, 47], classification of LADA as type 1 diabetes may contribute to a lower overall T1D-PGS in the present cohort. Furthermore, a limitation is the lack of valid time-to-diabetes information, as only secondary care diagnoses are captured in the registries, leaving prior diagnoses in primary care unobserved and thereby limiting time-to-event analyses.

Further research is necessary to validate our findings and assess the applicability of the T1D-PGS in women with GDM, ideally with prospective designs and larger cohorts comprising more women with type 1 diabetes.

In conclusion, the T1D-PGS demonstrated good predictive ability for development of type 1 diabetes in women with GDM, especially when combined with clinical risk factors during pregnancy. Furthermore, the T1D-PGS offers a possibility to distinguish between type 1 diabetes and other forms of diabetes, and thereby holds potential as a clinical tool to identify women who could benefit from targeted follow-up after pregnancy, potentially enabling therapeutic interventions to delay disease onset. However, several challenges remain for implementation into clinical practice, including defining thresholds for high risk and establishing standardised guidelines for clinical follow-up. The T2D-PGS showed no predictive ability for type 2 diabetes.

## Supplementary Information

Below is the link to the electronic supplementary material.

ESM Tables (PDF 245 KB)

## Data Availability

The data contain sensitive personal information and are thus not openly available due to data protection regulations. Access to the register data is governed and provided by Statistics Denmark and can be granted to researchers affiliated with authorised research institutions following approval from relevant Danish Authorities (Danish Data Protection Agency). Access to genetic and phenotypic data requires approval from the Danish Data Protection Agency and Scientific Ethical Committee and a data processor agreement must be signed between the data processor and data controller and may not be processed for purposes other than statistical and scientific studies.

## Abbreviations

AUC: Area under the curve
DNPR: Danish National Patient Registry
FPG: Fasting plasma glucose
GADA: Glutamic acid decarboxylase autoantibodies
GDM: Gestational diabetes mellitus
HLA: Human leukocyte antigen
ICD-10: The 10th revision of the International Classification of Diseases
IQR: Interquartile range
LADA: Latent autoimmune diabetes in adults
MODY: Maturity-onset diabetes of the young
OR: Odds ratio
PGS: Polygenic score
ROC: Receiver operating characteristic
SD: Standard deviation
T1D-PGS: PGS for type 1 diabetes
T2D-PGS: PGS for type 2 diabetes

## Authors’ relationships and activities

LR serves as the chairperson of the Diabetic Pregnancy Study Group (unpaid); has received grants from Novo Nordisk A/S (grant number: U1111-1209-6358) for the CopenFast trial, an investigator sponsored study, and from the Novo Nordisk Foundation (grant number: NNF24OC0091713) for an investigator sponsored randomised controlled trial; and participated in the European Association for the Study of Diabetes annual meeting 2023 as an invitee by Novo Nordisk. PD and ERM are involved in clinical studies on the use of insulin in pregnant women with pre-existing diabetes in collaboration with Novo Nordisk, but no personal honorarium is received. TH owns stocks in Novo Nordisk. The authors declare that there are no other relationships or activities that might bias, or be perceived to bias, their work.
