# Supplementary material for: Predicting future risk of type 1 and type 2 diabetes after gestational diabetes mellitus using polygenic scores: a cohort study
Source: Diabetologia. 2026 Aug 4;69(11):3261–72. doi: 10.1007/s00125-026-06824-0 (PMC13627188; doi:10.1007/s00125-026-06824-0)
Supplement: Supplementary file 1 [file 125_2026_6824_MOESM1_ESM.pdf]

Electronic Supplementary Material (ESM)

ESM Table 1. Cutoff values for the 3-h 50 g OGTT (1978-1985) and 3-h 75 g OGTT (1987-1996) used to diagnose GDM [6]

Time (minutes)	Venous plasma glucose (mmol/l)	
	50 g	75 g
0	6.4	6.2
30	10.1	10.9
60	10.1	11.1
90	8.7	9.2
120	7.6	8.9
150	7.6	8.2
180	6.6	7.3

GDM was diagnosed if ≥ 2 measurements exceeded the cutoff values.

Risk factor-based screening has remained standard in Denmark, but since 2004, GDM has been diagnosed based on a 2-h plasma glucose value ≥ 9 mmol/l following a 75 g OGTT, a threshold similar to the 2-h value (>8.9 mmol/l) of the 3-h 75 g OGTT.

ESM Table 2. Baseline characteristics according to diabetes development in women with previous gestational diabetes mellitus

Cumulative diabetes status a median of 30 years after index pregnancy[a]	**T1D (n=30)**	**T2D (n=157)**	**No DM (n=170)**
Pregnancy characteristics			
Maternal age at delivery (years)	29 (5)	32 (5)	32 (5)
Pregestational BMI (kg/m^2)	22.1 (20.2-24.2)	27.8 (23.7-33.0)	22.9 (20.4-26.5)
Family history of diabetes (yes)	8 (26.7)	75 (47.8)	69 (40.6)
Gestational age at GDM diagnosis (days)	217 (175-247)	227 (193-245)	236 (211-252)
Gestational age at delivery (days)	271 (260-275)	272 (265-277)	274 (270-279)
Fasting plasma glucose at GDM diagnosis (mmol/l)	5.7 (5.0-6.6)	5.3 (5.0-6.0)	4.8 (4.4-5.3)
Treatment			
- Diet	20 (66.7)	148 (94.3)	167 (99.4)
- Insulin	10 (33.3)	9 (5.7)	1 (0.6)
Clinical follow-up between 2000-2002 (median 11 years after index pregnancy)[b]			
Diabetes (self-reported and based on oral glucose tolerance test)			
- Yes	30 (100)	116 (73.9)	0 (0)
- No	0 (0)	41 (26.1)	170 (100)

Data are given as median (IQR), mean (SD), or n (%). Data available on >95% of participants. Characteristics of 13 women with MODY are not presented due to the low number of cases.

[a]Diabetes diagnoses obtained from both registry follow-up (median 30 years after pregnancy) and clinical follow-up (median 11 years after pregnancy).

[b]All women with type 1 diabetes had developed diabetes by the time of the clinical follow-up (median 11 years after pregnancy). Among women diagnosed with type 2 diabetes, 26.1% had not developed diabetes at the clinical follow-up and therefore developed diabetes in the subsequent period.

BMI, body mass index; GDM, gestational diabetes mellitus; No DM, no diabetes diagnosis; T1D, type 1 diabetes; T2D, type 2 diabetes.
